# Supplementary material for: Desensitisation of Notch signalling through dynamic adaptation in the nucleus
Source: EMBO J. 2021 Aug 16;40(18):e107245. doi: 10.15252/embj.2020107245 (PMC8441390; doi:10.15252/embj.2020107245)
Supplement: Supplementary file 2 — Expanded View Figures PDF [file EMBJ-40-e107245-s010.pdf]

Expanded View Figures

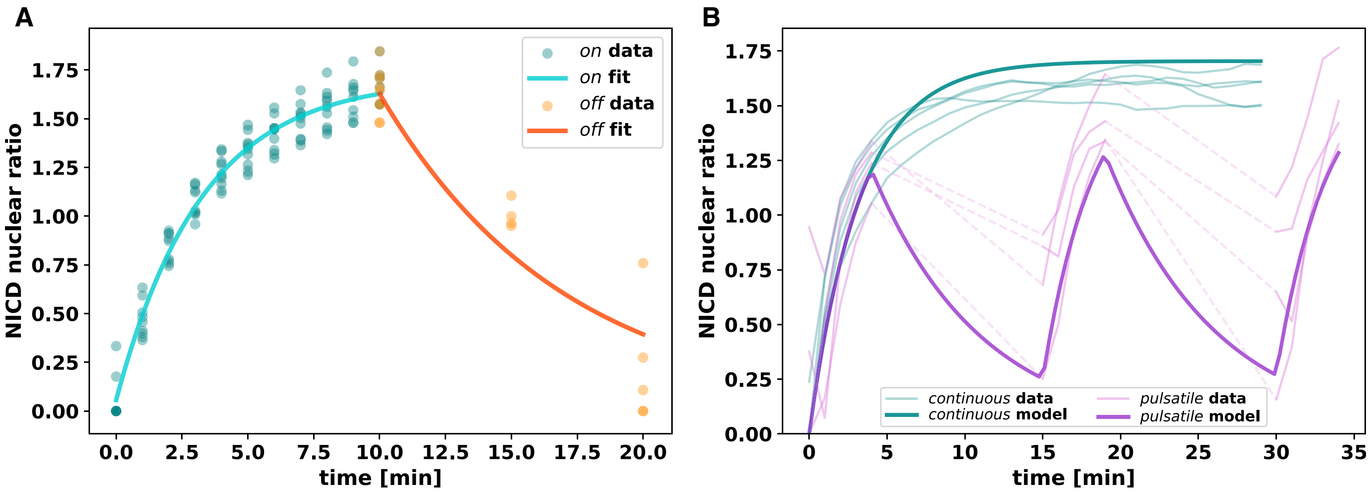

Figure EV1. Quantification and modelling of NICD nuclear translocation in response to opto-Notch activation.

A, B Experiments for the quantification and modelling of opto-Notch NICD nuclear import–export dynamics. Embryos expressed His::GFP in addition to the components of the optogenetic system as a nuclear counter-label, which was used for nuclear segmentation and quantification of mCherry::NICD. Raw data were bleaching-corrected with an exponential fit and shown is the nuclear/total ratio, which further normalises bleaching and embryo-to-embryo variability. (A) Quantification of import and export rates of mCherry::NICD. Embryos are first imaged under activating conditions for 10 min, then left in the dark for either 5 or 10 min before a final acquisition. Bold lines show the fit of a simple 2-compartment import–export ODE model. (B) NICD nuclear localisation during the standard continuous and pulsatile activation protocols, reproduced from Fig 2D and E. Strong lines are predictions from the 2-compartment import–export model, showing that they capture NICD translocation dynamics reasonably well. Remaining quantitative differences may be due to the limitations of bleaching correction on the data. See Materials and Methods for more details.

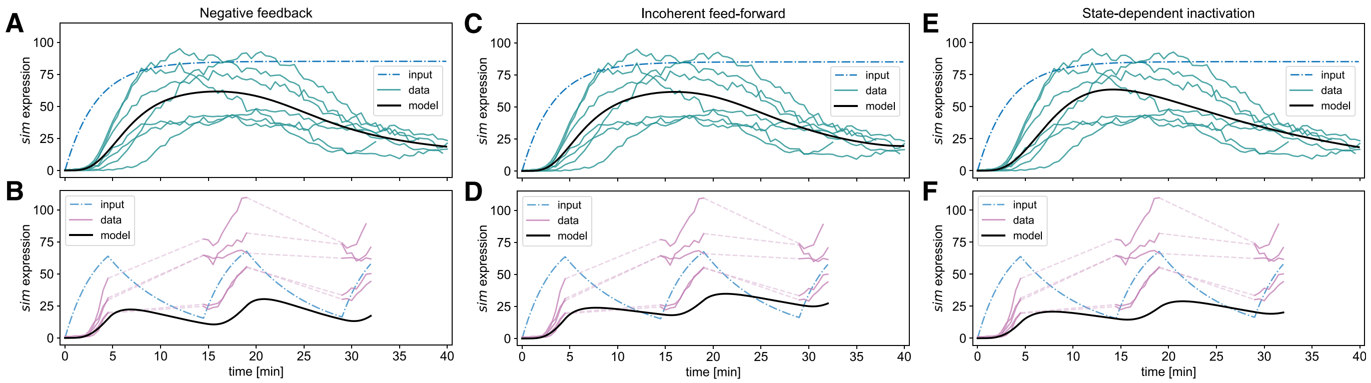

Figure EV2. Results of Hill-type ODE model fitting to *sim* adaptation dynamics.

A–F Simulation outcomes matching (Fig 3D–I), but using the more complex Hill-type models. (A, C, E) As expected, additional degrees of freedom allow for a more precise fit in non-linear areas of the *sim* expression profile. However, these models consistently underestimate *sim* expression in the pulsatile activation scenario (B, D, F).

© 2021 The Authors

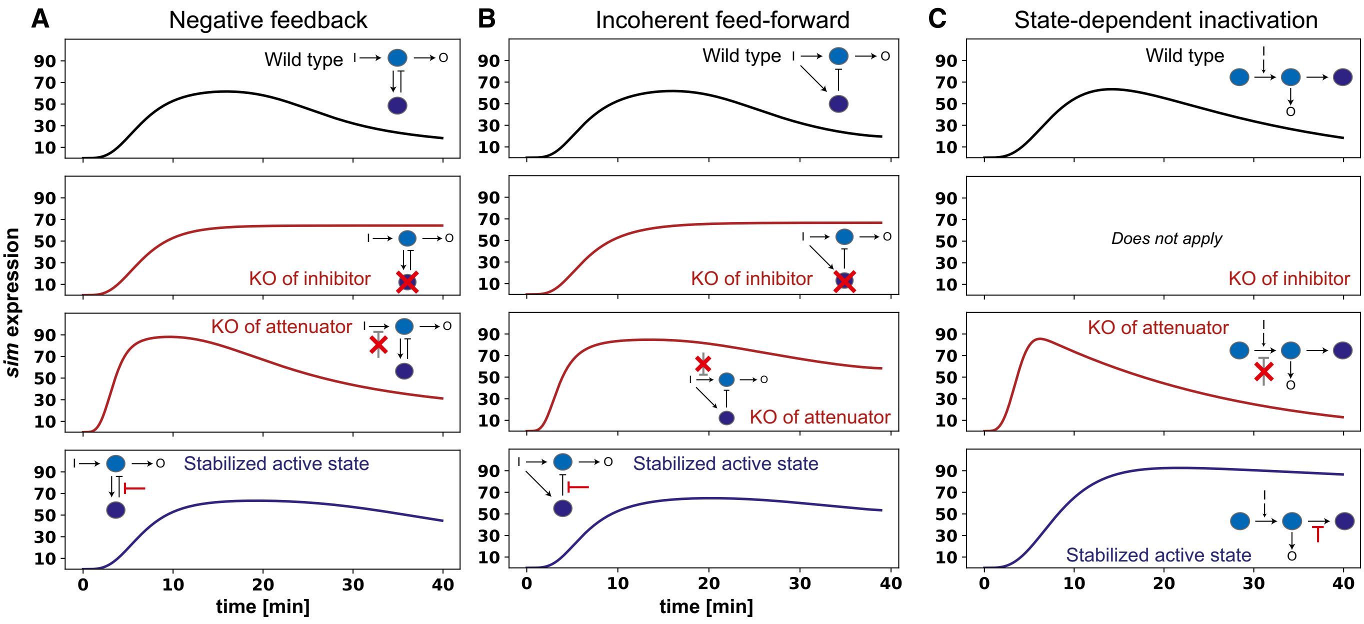

Figure EV3. Simulated responses to molecular perturbations for Hill-type ODE models.

A–C Simulation outcomes matching (Fig 4A–C), but using the more complex Hill-type models. Note that most outcomes remain very similar to their elementary mass-action counterparts. However, negative feedback shows some persistence of adaptation upon knock-out of an attenuator (A; row 3). Furthermore, only state-dependent inactivation shows an increase in *sim* expression upon stabilisation of (A_{on}), consistent with the results of Twist overexpression (Fig 4E).

 © 2021 The Authors

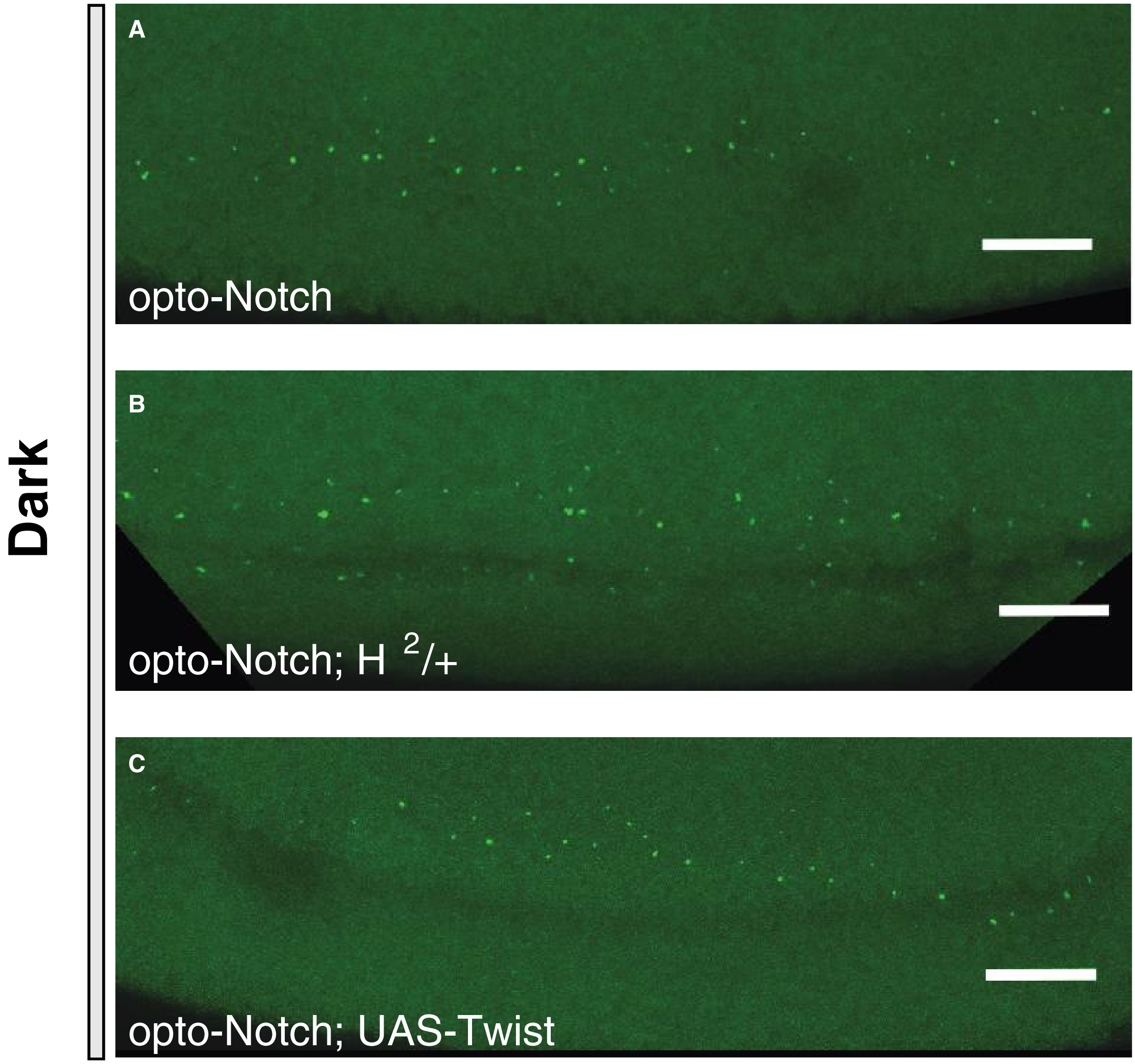

Figure EV4. *sim* expression in optogenetic embryos that developed in the dark.

A–C Maximum intensity z-projections (z = 25 μm) of embryos co-expressing opto-Notch, Mito-Zdk, MCP::GFP and *sim*-MS2 (A), or with the same genetic components as in (A) but heterozygous for the H^2 Hairless hypomorphic allele (B), or overexpressing UAS-Twist (C). Embryos were allowed to develop in the dark and imaged at the onset of gastrulation to record *sim* expression. Scale bars, 10 μm.

© 2021 The Authors

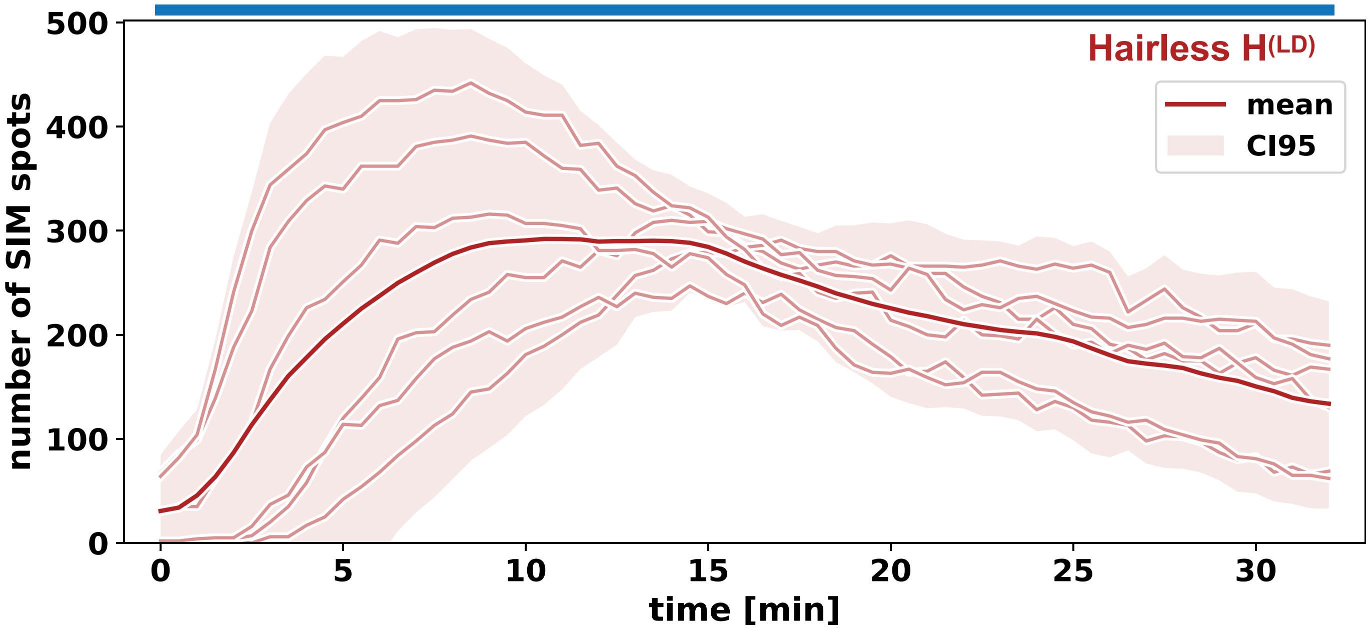

Figure EV5. Continuous activation experiments with non-binding Hairless allele.

Equivalent experiment to (Fig 4D), but with the Hairless allele $H^{(LD)}$, which cannot bind NICD's co-activator Su(H), confirming that Hairless loss-of-function increases the sensitivity of the *sim* promoter to NICD-driven activation but does not abolish adaptation.

 © 2021 The Authors
